# Successful surgical treatment of Stanford type A aortic dissection due to Salmonella aortitis

**DOI:** 10.1186/s13019-023-02318-x

**Published:** 2023-07-14

**Authors:** Shohei Yoshida, Adwaiy Manerikar, Mengou Zhu, Christopher Mehta

**Affiliations:** 1 Division of Cardiac Surgery, Bluhm Cardiovascular Institute, Northwestern University Feinberg School of Medicine and Northwestern Memorial Hospital, 676 North St. Clair, Suite 7-300, Chicago, IL 60611 USA; 2 Division of Internal Medicine, Northwestern University Feinberg School of Medicine and Northwestern Memorial Hospital, Chicago, IL USA

**Keywords:** Salmonella aortitis, Stanford type A aortic dissection

## Abstract

**Background:**

*Salmonella spp.* cause infectious aortitis through the hematogenous spread of an intestinal Salmonella infection. Salmonella aortitis can result in extensive tissue damage in the aorta leading to complications including dissection, abscess formation, pseudoaneurysms, and rupture, which require early diagnosis and treatment with both surgery and antibiotic therapy.

**Case presentation:**

We report a case of Salmonella aortitis complicated by Stanford type A aortic dissection. A 62-year-old man with a history of heroin use presented with chest pain, epigastric pain and vomiting. The computed tomography scan showed Stanford type A aortic dissection without malperfusion. At the time of surgery, an aortic dissection with purulent fluid and contained rupture was noted in the ascending aorta. Fluid culture was consistent with Salmonella. A composite valve-graft conduit aortic root replacement with ascending aorta and hemiarch replacement was performed. The patient recovered well and was discharged on long-term antibiotics.

**Conclusions:**

This rare case of a Stanford type A aortic dissection with contained rupture due to Salmonella aortitis was successfully treated with emergent surgery and antibiotic therapy.

## Background

Infectious aortitis is a rare but serious clinical condition that is highly lethal if treatment is delayed. It is more likely to occur in aortic tissue with prior vascular disease such as atherosclerosis and aneurysm [1]. About 60% of infectious aortitis cases are caused by gram-positive organisms, but gram-negative organisms such as *Salmonella spp.* are also frequent causative organisms [2]. In immunocompetent adults, *Salmonella spp.* is most commonly a foodborne infection that results in acute gastroenteritis, but extraintestinal infection such as infectious aortitis can occur through hematogenous spread in patients with certain risk factors [3]. While *Salmonella spp.* is more likely to be implicated in infections of the abdominal aorta, it can also cause infectious aortitis of the thoracic aorta and result in serious complications [2]. We report a case of a 62-year-old patient with thoracic aortitis caused by *Salmonella spp.* complicated by Stanford type A aortic dissection.

## Case presentation

**Fig. 1:**
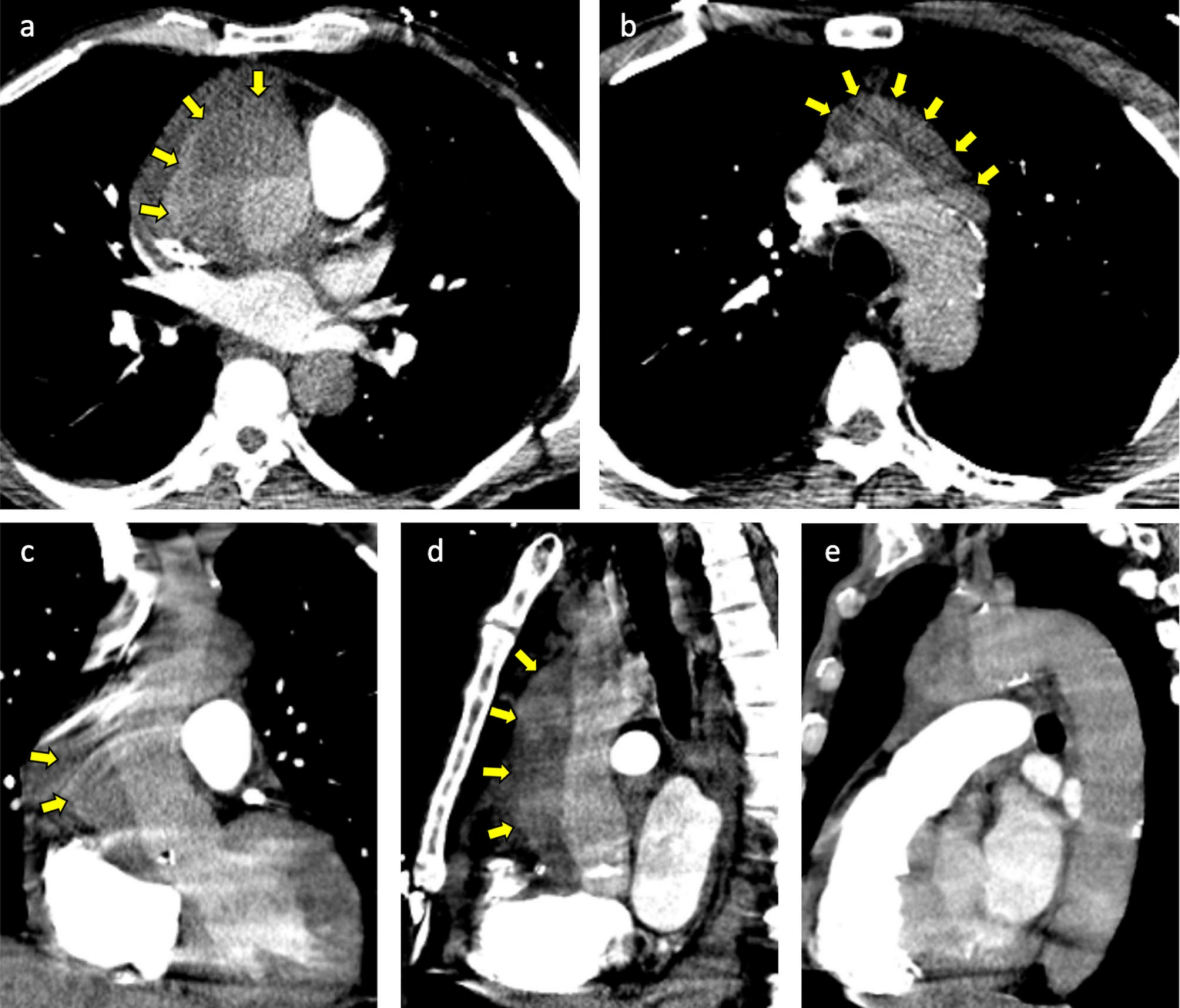
Preoperative computed tomography shows Stanford type A aortic dissection of the ascending aorta and the aortic arch (arrows). There was no dissection in the descending aorta. (**a**) Transverse view of the ascending aorta, (**b**) transverse view of the aortic arch, (**c**) coronal view of the ascending aorta, (**d**) sagittal view of the ascending aorta, and (**e**) sagittal view of the descending aorta

**Fig. 2:**
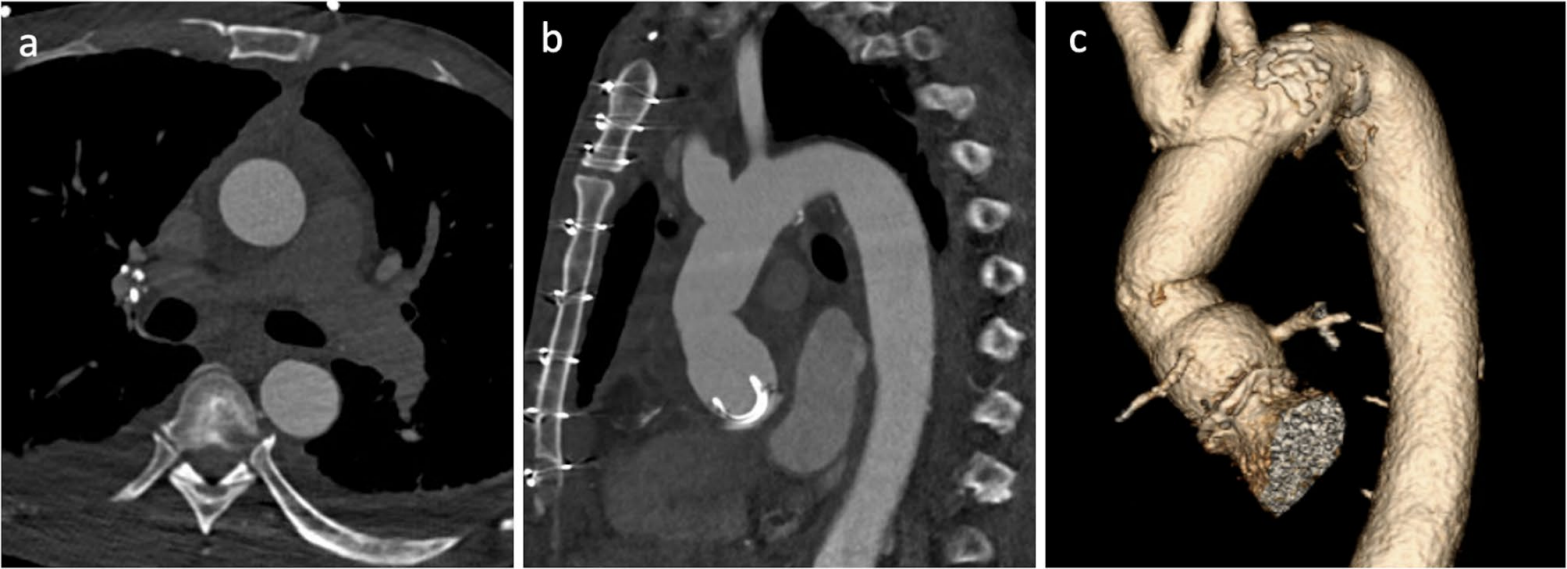
(**a**) Transverse view of postoperative computed tomography shows reconstructed ascending aorta. (**b**) Sagittal view of postoperative computed tomography shows reconstructed aortic root and ascending aorta. (**c**) Reconstructive computed tomography angiogram shows reconstructed aortic root and ascending aorta with decent proximal coronary artery enhancement

The patient was a 62-year-old man with a past medical history significant for hypertension, hyperlipidemia, heroin use, chronic obstructive pulmonary disease, and subdural hematoma 2 months prior who presented to an outside hospital with symptoms of chest pain, epigastric pain and vomiting for the past day. The systolic blood pressure was less than 120 mmHg and there was no difference in blood pressure between extremities. Distal pulses were palpable in all extremities. The patient was afebrile, and the abdomen was flat and soft on physical examination. The blood test revealed 19.2 × 10^3^/µL of white blood cell count and elevated D-dimer levels. The electrocardiography showed sinus rhythm without ST elevation. A pulmonary embolism was suspected, and non-gated computed tomography with contrast during the pulmonary artery phase was performed. The computed tomography showed no evidence of pulmonary embolism, but instead demonstrated a Stanford type A aortic dissection involving the ascending aorta and aortic arch, with a dilated aortic root and ascending aorta, although it appeared atypical due to the enhancement at the pulmonary artery phase (Fig. 1). This was classified as DeBakey type I. The patient was transferred to our center for emergent aortic dissection surgery. The systolic blood pressure had been kept less than 120 mmHg and the heart rate less than 60 bpm without medications during the transfer. After induction of anesthesia, transesophageal echocardiography was performed which showed aortic dissection in the aortic sinus, sinotubular junction, mid and distal ascending aorta, and mid arch. There was a large hematoma in the mid and distal ascending aorta. Also, normal biventricular function without valvular dysfunction except moderate aortic regurgitation was observed. The aortic valve was tricuspid, but there was no evidence of abscess, fistula, or rupture on the transesophageal echocardiography. After full sternotomy, cardiopulmonary bypass was established by right axillary artery cannulation and femoral venous cannulation. There was dense inflammatory tissue surrounding the ascending aorta as well as the entire heart with unclear tissue planes. The aorta was cross-clamped and the ascending aorta divided. An aortic dissection with purulent fluid from the false lumen and contained rupture was noted in the mid-ascending aorta. The purulent fluid was swabbed and sent for culture. An entry tear was identified in the distal ascending aorta, which extended proximally into the aortic root including aortic sinus and proximal right coronary artery. No entry tears were noted in the aortic arch. After resecting the aortic wall that was thought to be infected, thorough irrigation was performed. The resected ascending aortic wall was then sent for pathological examination. Due to dilation and dissection of the aortic root and moderate aortic regurgitation with thickening and ulcerated calcifications in the non-coronary cusp, the aortic root was replaced with a resilient bovine pericardial valved conduit and coronary arteries were reimplanted as buttons in a Bentall technique. We performed ascending aorta and hemiarch replacement, excluding the entry tear. The distal aortic anastomosis was performed at the dissection site and reinforced with an external felt strip under deep hypothermia and circulation arrest with antegrade cerebral perfusion. The pathological examination demonstrated an intimal layer with multiple atheromatous plaques separating from the adventitial layer with fibrous thickening. Figure 2 shows the reconstructed ascending aorta and aortic arch by computed tomography with contrast performed 5 days after surgery. Vancomycin was continued post-operatively for high suspicion of gram-positive cocci infection and subsequently, cefepime was initiated due to intraoperative tissue culture growing gram-negative organisms. Following subsequent speciation of cultures to to pan-susceptible Salmonella, the antibiotic regimen was further narrowed to ceftriaxone. The postoperative course was unremarkable, and the patient was discharged on long-term antibiotics on postoperative day 15. We confirmed the patient survived for 30 days after the surgery.

## Discussion

Infectious aortitis can be a serious consequence of systemic Salmonella infection. In the United States, Salmonella infection is mostly caused by non-typhoid species and is often acquired from undercooked poultry and egg, as well as contaminated dairy products [3–5]. While *Salmonella spp.* most commonly result in self-limited gastroenteritis, almost all serotypes of *Salmonella spp. *have the potential of invading the intestinal barrier. As a result, some patients may develop bacteremia and eventually extraintestinal infection through hematogenous spread [5]. Bacteremia and extraintestinal manifestations occur in about 5% of patients with *Salmonella spp.* infection, and it is more likely to develop in patients with predisposing factors such as immunocompromised status, diabetes, chronic renal and liver disease, and abnormal gastrointestinal barrier [1, 3–6]. When implicated in infectious aortitis, *Salmonella spp.* is most likely to be seeded in the abdominal aorta due to its proximity to the intestines [2]. In this patient, his history was notable for extensive heroin use via multiple routes, including intravenous, oral, and sublingual injections, which is a less common risk factor that drastically increases the chance of inoculating *Salmonella spp.* from the saliva into the blood. The atypical means of acquiring systemic Salmonella infection may also explain his atypical site of infection in the ascending aorta.

Given its invasive nature, *Salmonella spp.* are more likely to cause tissue damage in the aortic wall and result in structural complications such as dissection, pseudoaneurysm, rupture, and purulent abscess formation [7, 8]. This is consistent with the intraoperative findings of our patient: he was found to have an aortic dissection with purulent fluid complicated by contained rupture, extending into the aortic root and right coronary artery. The high likelihood of structural complications makes early imaging a crucial component in diagnosing infectious aortitis, and imaging may have higher diagnostic value than clinical symptoms and labs [7, 8]. Classic symptoms such as high fevers and leukocytosis are not sensitive or specific for infectious aortitis, and blood cultures are only positive in 50-70% of patients with infectious aortitis, especially if the causative organism is a gram-negative rod [9]. It is noteworthy that our patient was brought to the operating room due to imaging findings of Stanford type A aortic dissection, and the diagnosis of infectious aortitis was made intraoperatively based on the grossly inflammatory aortic tissue, the presence of purulent fluid, and positive tissue culture, which suggested the aortic dissection might not be acute. The patient never presented with fevers during his admission, and his blood cultures remained negative. There were no specific results indicating Salmonella-infected aortic disease found in either the pathological or preoperative examination. In this patient, early surgery based on early imaging evidence of dissection was both diagnostic and therapeutic for Salmonella aortitis and is the key to management.

Antibiotic treatment and surgery are two key components of Salmonella infectious aortitis management. Management with antibiotics alone without surgery results in a mortality of greater than 90%, while the combination of surgery and antibiotics can reduce the mortality to 40%, regardless of what kind of structural complication is present [10]. In our case presentation, the patient received an emergent composite valve-graft conduit aortic root replacement with ascending aorta and hemiarch replacement given his Stanford type A aortic dissection. Several classic methods have been reported as being potentially effective in preventing risks of postoperative infection, namely omentopexy, rifampicin-gelatin artificial graft replacement, and allograft aortic root replacement. Each of these methods have their advantages and limitations. Rifampicin-gelatin grafts have been shown to reduce the chance of postoperative infection in several studies, however most clinical data has focused on the efficacy in the prevention of methicillin-susceptible *Staphylococcus aureus* (MSSA) and methicillin-susceptible *Staphylococcus epidermidis* (MSSE) [11–14]. While rifampicin can also be effective against gram-negative organisms such as *Salmonella spp.*, higher systemic antibiotic concentrations are often required to achieve therapeutic goal, which significantly limits the application of such grafts to our patient’s case [15]. Allograft aortic root replacement is another classic method to prevent postoperative infection [16, 17]. While most often implemented as treatment for congenital heart disease, aortic root allograft surgery has been used for treatment of aortic valve endocarditis and and has also been shown to be effective in reducing postoperative infection risks [18]. While there has been debate on the durability of cryopreserved allografts, more recent studies have found that aortic allografts have comparable durability to prosthetic valves and can last for more than 15 years with careful selection of recipients and newer implantation techniques [19, 20]. However, underlying thoracic aortic disease is often associated with structural complications, which limits the applicability of allografts in treating type A aortic dissection and thoracic aortitis [20]. Moreover, the availability of properly sized allografts also limits its use in emergent surgical cases. Omentopexy is another method that has been shown to reduce or prevent the occurrence of postoperative infection after aortic graft surgery [21, 22]. The omental flap serves as a physical barrier to prevent recurrent infection of the implanted graft, and the rich blood supply to the omental tissue can also facilitate clearance of bacteria [21]. However, the clinical value of omentopexy may not be applicable to our case because the aortitis is most likely the result of hematogenous spread of bacteria inoculated through IV and sublingual drug use, as opposed to contiguous spread in most cases of Salmonella aortitis. In Salmonella aortitis patients with lower surgical risks, such as patients without acute dissection, patients without uncontrolled sepsis, and patients with a lack of evidence of abscess or gross purulence, endovascular approaches may be acceptable [23, 24]. In terms of antibiotic therapy, there is no specific guideline on the empirical choice of regimen and duration of therapy for Salmonella aortitis. However, empirical coverage with a third-generation cephalosporin is commonly used, and most providers agree with an extended course of intravenous antibiotics for 6–8 weeks. Some providers may even recommend life-long oral suppression therapy [4, 25–27]. In our case report, the intraoperative tissue culture demonstrated *Salmonella spp.*, for which the antibiotic regimen was narrowed to ceftriaxone from vancomycin and cefepime. At least six weeks of antibiotic therapy was recommended. Oral trimethoprim-sulfamethoxazole as a salvage therapy could be an option if a patient refuses long-term intravenous antibiotics.

## Conclusion

Salmonella spp. can cause infectious aortitis, which is a rare condition. However, when it occurs, it can lead to extensive tissue damage in the aorta and give rise to complications such as dissection, abscess formation, pseudoaneurysms, and rupture. These complications require early detection and treatment through both surgery and antibiotic therapy. In this case, a Stanford type A aortic dissection with contained rupture caused by Salmonella aortitis was effectively treated with emergent surgery and antibiotic therapy.

## Data Availability

Data sharing is not applicable to this article as no datasets were generated or analysed during the current study.
